# Multiple functions of precursor BDNF to CNS neurons: negative regulation of neurite growth, spine formation and cell survival

**DOI:** 10.1186/1756-6606-2-27

**Published:** 2009-08-13

**Authors:** Hisatsugu Koshimizu, Kazuyuki Kiyosue, Tomoko Hara, Shunsuke Hazama, Shingo Suzuki, Koichi Uegaki, Guhan Nagappan, Eugene Zaitsev, Takatsugu Hirokawa, Yoshiro Tatsu, Akihiko Ogura, Bai Lu, Masami Kojima

**Affiliations:** 1 National Institute of Advanced Industrial Science and Technology (AIST), Ikeda, 563-8577 Japan; 2 Core Research for Evolutional Science and Technology (CREST), Japan Science and Technology Agency (JST), Kawaguchi, 332-0012, Japan; 3 Department of Neuroscience, Osaka University Graduate School of Frontier Biosciences, Toyonaka, 560-0043 Japan; 4 Section on Neural Development and Plasticity, The *Eunice Kennedy Shriver *National Institute of Child Health and Human Development (NICHD), National Institutes of Health (NIH), Bethesda, MD, 20892 USA

## Abstract

**Background:**

Proneurotrophins and mature neurotrophins elicit opposite effects via the p75 neurotrophin receptor (p75^NTR^) and Trk tyrosine kinase receptors, respectively; however the molecular roles of proneurotrophins in the CNS are not fully understood.

**Results:**

Based on two rare single nucleotide polymorphisms (SNPs) of the *human brain-derived neurotrophic factor (BDNF) *gene, we generated R125M-, R127L- and R125M/R127L-BDNF, which have amino acid substitution(s) near the cleavage site between the pro- and mature-domain of BDNF. Western blot analyses demonstrated that these BDNF variants are poorly cleaved and result in the predominant secretion of proBDNF. Using these cleavage-resistant proBDNF (CR-proBDNF) variants, the molecular and cellular roles of proBDNF on the CNS neurons were examined. First, CR-proBDNF showed normal intracellular distribution and secretion in cultured hippocampal neurons, suggesting that inhibition of proBDNF cleavage does not affect intracellular transportation and secretion of BDNF. Second, we purified recombinant CR-proBDNF and tested its biological effects using cultured CNS neurons. Treatment with CR-proBDNF elicited apoptosis of cultured cerebellar granule neurons (CGNs), while treatment with mature BDNF (matBDNF) promoted cell survival. Third, we examined the effects of CR-proBDNF on neuronal morphology using more than 2-week cultures of basal forebrain cholinergic neurons (BFCNs) and hippocampal neurons. Interestingly, in marked contrast to the action of matBDNF, which increased the number of cholinergic fibers and hippocampal dendritic spines, CR-proBDNF dramatically reduced the number of cholinergic fibers and hippocampal dendritic spines, without affecting the survival of these neurons.

**Conclusion:**

These results suggest that proBDNF has distinct functions in different populations of CNS neurons and might be responsible for specific physiological cellular processes in the brain.

## Background

The development and functioning of the mammalian nervous system are regulated by neurotrophins, that are a family of neurotrophic factors which includes nerve growth factor (NGF), BDNF, neurotrophin-3 (NT-3), and NT-4/5 [1]. Like many peptide hormones and growth factors, neurotrophins are first synthesized as precursors and are subsequently cleaved either intracellularly by prohormone convertases (PCs) and/or furin, or extracellularly by plasmin and matrix metalloproteases (MMPs) to form mature proteins [2]. These proteins elicit their biological actions by binding to the Trk family of receptor-type tyrosine kinases [3]. For decades, proneurotrophins were thought to be biologically inactive; the dogma was changed when Lee *et al*. [4] reported that proNGF preferentially interacted with p75^NTR ^instead of Trk receptors. This interaction leads to apoptosis of peripheral neurons, an effect opposite to the pro-survival action of mature NGF. Since then, proapoptotic effects of proNGF and proBDNF mediated by p75^NTR ^have been demonstrated in a number of model systems [4-8]. Therefore, proteolytic cleavage of proneurotrophins is thought to be an important regulatory step for the direction of neurotrophin function, given the diametrically opposed functions of proneurotrophins and mature neurotrophins, which are elicited via the p75^NTR ^and Trk receptors, respectively [9,10].

**Figure 1:**
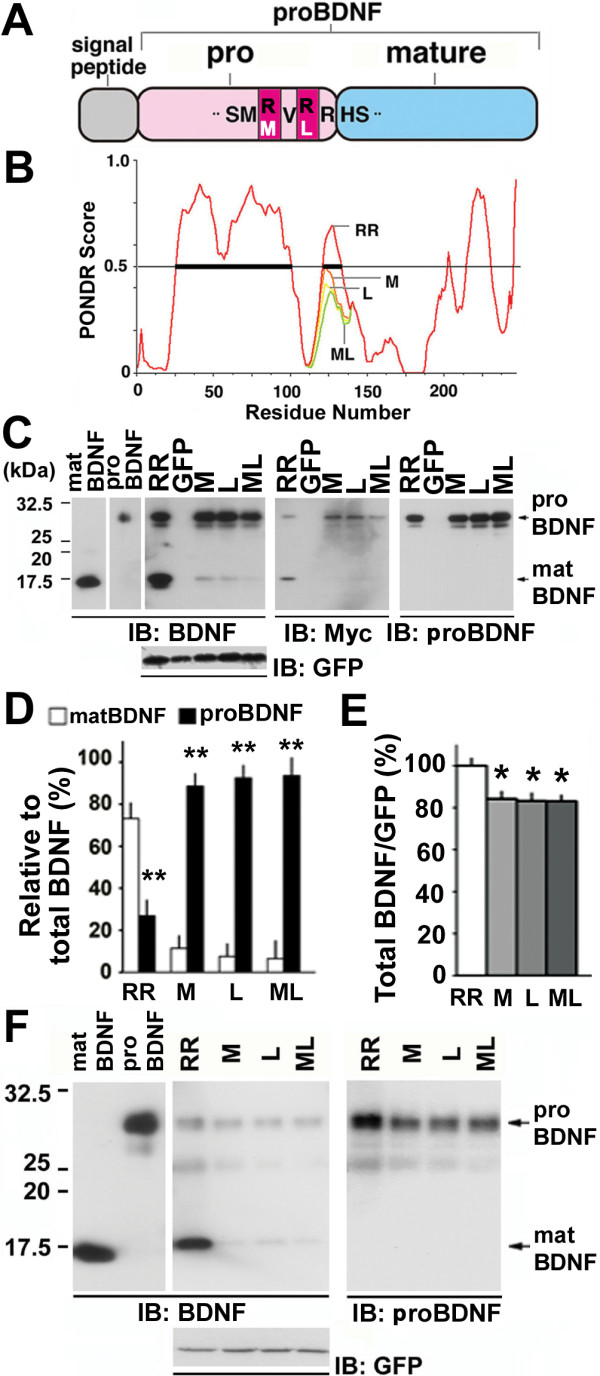
**BDNF polymorphic substitutions inhibit the conversion of proBDNF to matBDNF and lead to predominant secretion of proBDNF in CNS neurons**. (A) Schematic of human BDNF protein. Amino acid substitutions near the cleavage site in rare SNP variants are represented by single-letter symbols. The amino acid substitutions caused by the SNPs are depicted by white symbols. (B) Bioinformatic prediction of changes in the secondary structure of proBDNF SNPs variants using PONDR. M, L and ML depict the amino acid substitution of R125M, R127L and R125M/R127L, respectively. The disordered regions (PONDR score > 0.5) of wild-type BDNF (aa 25–103 and 120–140) is highlighted by bold lines. (C) Inhibition of the intracellular cleavage of SNP variants in cultured hippocampal neurons. The cultures were infected with Sindbis viruses expressing wild-type (RR), R125M (M), R127L (L), and R125M/R127L (ML) constructs for 12 h and 3 days later processed for Western blot analysis using anti-BDNF (IB: BDNF) and anti-Myc antibodies (IB: Myc). Doublet bands of 29- and 32-kDa were detected with the monoclonal anti-proBDNF antibody (IB: proBDNF), whereas an additional band of 14-kDa (matBDNF) was detected using an antibody against the mature domain (IB: BDNF). The levels of bicistronically expressed GFP, which correlated with the viral infection levels, were similar in all cultures. Note that proBDNF bands were predominant in the lysates of cells expressing the M, L, and ML constructs, while matBDNF expression was residual. In C and F, 1 ng recombinant matBDNF and proBDNF (R125M/R127L-BDNF) were used as positive controls. (D) The ratio of proBDNF to matBDNF was quantified by densitometric analysis on BDNF, and proBDNF bands. **P < 0.01 (Student's *t*-test) when compared with matBDNF (matBDNF_RR _+ proBDNF_RR _= 100% as control). n = 3 independent experiments. (E) The entire amount of BDNF was quantified by densitometric analysis on matBDNF, proBDNF, and GFP bands. *P < 0.05 (*t*-test) when compared with RR (100% as control). n = 3 independent experiments. (F) Secretion of poorly cleaved proBDNF. Cultured cerebral cortical neurons were incubated with Sindbis viruses for 12 h and maintained in serum-containing medium for 3 days. Supernatants were collected and immunoprecipitated using anti-Myc antibody-conjugated agarose beads, followed by Western blot analysis using rabbit antibody against the mature domain (IB: BDNF) and mouse antibody recognizing the prodomain (IB: proBDNF). Cell lysates were blotted with anti-GFP antibody to normalize viral infection (IB: GFP).

**Figure 2:**
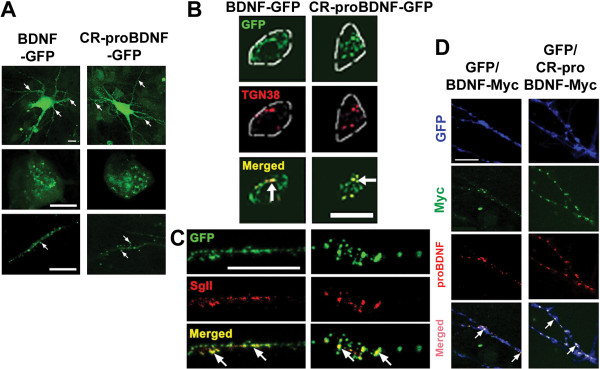
**Intracellular proteolytic cleavage of proBDNF is not a crucial step for intracellular transportation and secretion of BDNF in hippocampal neurons**. (A) Distribution of GFP-tagged wild-type BDNF (BDNF-GFP) and cleavage-resistant (CR)-proBDNF (R125M/R127L-BDNF-GFP) in the cell body and processes. Cultured neurons were subjected to Sindbis viruses expressing the indicated constructs for 3 h. Three days later, the neurons were fixed for imaging of GFP fluorescence using confocal microscope. Representative low-magnification (upper) and high-magnification images of the cell body (middle) and neuronal processes (bottom) are shown. (B-C) The infected cells were immunostained with the indicated antibodies for confocal imaging. Note that CR-proBDNF-GFP greatly co-localized with TGN38 (B) and SgII (C), similarly to wild-type BDNF-GFP (arrows). Scale bar in all images, 10 μm. (D) Expression of wild-type proBDNF-Myc or CR-proBDNF-Myc. Hippocampal neurons were introduced with constructs encoding GFP and wild-type BDNF-Myc or CR-proBDNF-Myc using Sindbis virus expression system. Three-days after a brief (3 h) infection, cells were double-stained using anti-Myc and anti-proBDNF antibodies. BDNF-Myc signals largely co-localized with proBDNF signals (arrows) indicating that proBDNF is the predominant intracellular isoform. Scale bar, 5 μm.

The cleavage of proBDNF (^125^RVRR^128^↓ HS in Fig. 1A), [2] is thought to take place either intracellularly by serine proteases such as PC1/3 and/or furin, or extracellularly by extracellular proteinases such as plasmin activated by tissue plasminogen activator (tPA), and/or MMPs. The importance of proBDNF cleavage has been reported recently [11,12]. These two reports suggest that proBDNF cleavage in the nervous system is regulated in a more specific manner and depends on the cellular context.

However, little is known about the function of proBDNF when compared with the numerous reports on the mechanisms of mature BDNF (matBDNF) action [10,13]. *In vitro *experiments have shown that proBDNF enhances apoptosis of sympathetic neurons and basal forebrain neurons [7,8,14]. Application of exogenous proBDNF facilitates long-term depression (LTD), through an interaction with p75^NTR ^[15], suggesting that proBDNF may exert multiple biological actions in the nervous system.

Another important question is whether proBDNF has a physiological function [10,13]. The two rare human SNPs at nucleotides 373 (G/T) and 379 (G/T) (Reference SNP cluster id: rs1048220 and rs1048221, respectively) present in the coding region of the human BDNF gene are located near the cleavage site (Fig. 1A) and theoretically lead to the substitution of the arginine residues (R) by methionine (R125M) and leucine (R127L), respectively (Fig. 1A). Although the precise validation for these SNPs has not been fully determined, cDNA analysis on R125M suggests that one out of 92 persons from Utah State carried M-type BDNF gene . Thus, these polymorphisms may affect the cellular mechanisms and biological functions of BDNF in human brain.

In the present study, taking advantage of the cleavage resistance of these BDNF variants (CR-proBDNF), various cultured CNS neurons including hippocampal neurons, CGNs, and BFCNs were treated with recombinant CR-proBDNF protein to address the molecular functions of proBDNF on CNS neurons. Our studies suggested that CR-proBDNF showed normal intracellular distribution and secretion in cultured hippocampal neurons and that CR-proBDNF exerts distinct functions in cerebellar granule neurons (CGNs), basal forebrain cholinergic neurons (BFCNs) and hippocampal neurons.

## Results

### A bioinformatic analysis predicted SNPs-dependent changes in the secondary structure of proBDNF

In an initial attempt to study the effects of the rare SNPs (Fig. 1A), we analyzed the secondary structure of the human BDNF protein corresponding to the region containing SNPs using a bioinformatic tool, Predictor of Natural Disordered Regions algorithm (PONDR) [16]. The tool, which predicts the disordered regions in the proteins, identified regions corresponding to amino acids (aa) 25–103 and aa 120–140 to be disordered (Fig. 1B, PONDR score > 0.5). Consistent with the X-ray structure analysis [17], mature domain of BDNF (residue 129–247) was predicted to be thermally stable. However, when the human SNP mutations (Fig. 1A, R125M and R127L) were introduced, the PONDR score, and hence the degree of disorder, decreased markedly (Fig. 1B, aa 120–140, M, L and ML) suggesting that the region encompassing the protease cleavage site may undergo disordered-to-ordered transition when associated with other proteins, such as the proteases [16]. This result raises the possibility that the amino acid substitutions caused by SNPs may affect the molecular interaction of proBDNF with proteases and consequently interfere with the cleavage of proBDNF itself.

### R125M-, R127L-, and R125M/R127L-BDNF SNP variants are poorly cleaved in hippocampal neurons

We next introduced wild-type BDNF (RR) or the BDNF variants: R125M (M), R127L (L), or R125M/R127L (ML), which were tagged with a Myc epitope at the C-terminus [Additional file 1A], into cultured hippocampal neurons using Sindbis virus infection [18]. To normalize for the efficiency of gene transfer, we used a bicistronic vector that expressed both BDNF and green fluorescent protein (GFP) [19]. The multiplicity of infection (MOI) was 10 and 1 for Western blotting and immunostaining, respectively [18]. Such precise controls of Sindbis virus infection did not promote cell death of the neurons [18]. Western blotting was performed to compare the processing of these exogenous proteins. In control neurons expressing BDNF_RR_, we detected a doublet band of 29- and 32-kDa (proBDNF) and a 14-kDa band (matBDNF), using either anti-BDNF or anti-Myc antibodies (RR in Fig. 1C, IB: BDNF and Myc). Exogenously expressed BDNF_RR _appeared to be cleaved in a fashion similar to endogenous BDNF [20]. Interestingly, the proBDNF bands (29- and 32-kDa) were predominant in neurons expressing BDNF_M_, BDNF_L_, or BDNF_ML_, which showed residual levels of matBDNF (14-kDa) (M, L, and ML in Fig. 1C, IB: BDNF and Myc). This finding was confirmed using a quantitative analysis (Fig. 1D). Bands corresponding to precursor BDNF (Fig. 1C, IB: proBDNF) were also detected using anti-prodomain monoclonal antibody [Additional file 1B, mouse]. We ascertained that the apparent difference in the ratio of proBDNF to matBDNF between the wild-type and BDNF variants was due to differences in proteolytic processing and not to variability in the efficiency of Sindbis virus infection or BDNF expression levels, as all samples exhibited similar levels of GFP expression from the bicistronic vector used in this study (Fig. 1C). However, the total amount of BDNF {(matBDNF + proBDNF)/GFP} was slightly, yet significantly, reduced in neurons expressing the mutant BDNF constructs (Fig. 1E), which suggests that inhibition of proBDNF cleavage may affect the overall levels of expression of BDNF. These results showed that the amino acid substitutions associated with the SNPs (R125M, R127L, and R125M/R127L) inhibit the proteolytic cleavage of proBDNF to matBDNF in CNS neurons.

To examine whether the cleavage resistance affected BDNF secretion, we expressed the constructs encoding Myc-tagged BDNF_RR_, BDNF_M_, BDNF_L_, or BDNF_ML _in cultured cortical neurons using the transfection protocol depicted in Fig. 1C. Supernatants collected 3 days later were immunoprecipitated using an anti-Myc antibody conjugated to agarose beads. Secreted BDNF isoforms were detected by Western blot analysis using an anti-mature domain antibody that detects both proBDNF and matBDNF (Fig. 1F). Neurons expressing wild-type BDNF (RR) secreted several BDNF species: 30- and 32-kDa precursor forms, a 26-kDa intermediate form, and a 14-kDa mature form (RR in Fig. 1F, IB: BDNF). In contrast, supernatants collected from neurons expressing the BDNF polymorphic variants (M, L, and ML) contained predominantly the precursor forms with negligible, but detectable, levels of matBDNF (M, L, and ML in Fig. 1F, IB: BDNF). These results were confirmed using an anti-prodomain monoclonal antibody (M, L, and ML in Fig. 1F, IB: proBDNF). These results suggest that the BDNF polymorphic variants markedly inhibit the conversion of proBDNF to matBDNF, either intracellularly or extracellularly, and that they are mainly secreted as proBDNF.

### Inhibition of proBDNF cleavage does not affect intracellular transportation and secretion of BDNF in hippocampal neurons

Previous studies suggest that inhibition of intracellular cleavage by furin alters the subcellular distribution and secretion of neurotrophins, particularly of NT-3 [21]. To determine whether the two SNPs affect the intracellular distribution of BDNF, we expressed transgenes encoding GFP-tagged (C-terminus) wild-type BDNF (BDNF-GFP) or cleavage-resistant R125M/R127L-BDNF (CR-proBDNF) in cultured hippocampal neurons according to a previous report [18] (Fig. 2A). The two fusion proteins exhibited similar distribution patterns in the cell body and neuronal processes (Fig. 2A, arrows). Immunocytochemical analysis demonstrated that both BDNF-GFP and CR-proBDNF-GFP colocalized with a trans-Golgi network marker, TGN38 (Fig. 2B, arrows) and a secretory vesicle marker, secretogranin II (SgII) (Fig. 2C, arrows). An immunostaining study further showed that the signals of BDNF-Myc or CR-proBDNF-Myc were largely colocalized with the proBDNF signals (Fig. 2D, arrows). These findings, together with the result of Fig. 1F, suggest that inhibition of BDNF cleavage by the amino acid substitutions did not greatly influence the subcellular distribution of BDNF and that intracellular cleavage of proBDNF is not a critical step for intracellular transportation and secretion of BDNF in hippocampal neurons.

### Recombinant CR-proBDNF elicits apoptosis of cerebellar granule neurons cultured in low K^+ ^containing medium

**Figure 3:**
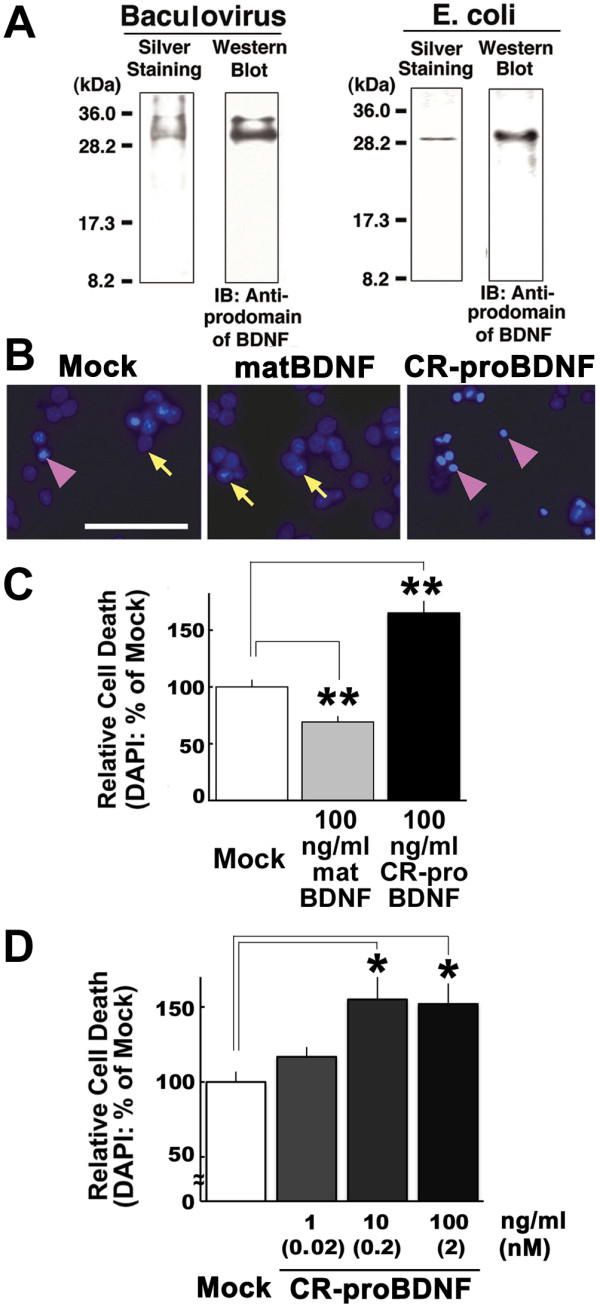
**Recombinant CR-proBDNF induces apoptosis of cerebellar granule neurons cultured in low K^+^-containing medium**. (A) Preparation of recombinant CR-proBDNF (R125M/R127L-BDNF: proBDNF_ML_) using the Baculovirus or *E. coli *expression systems. Protein products were analyzed by silver staining (left) and Western blotting using rabbit antibody against BDNF prodomain. (B-C) Opposite actions of CR-proBDNF and matBDNF revealed by apoptosis assay. After 4 days of culture in HK medium, CGNs were treated with *E. coli*-derived CR-proBDNF or matBDNF in LK medium. Cell death was assessed using DAPI staining 48 h after treatment with the indicated drug in LK medium. (B) Arrows and arrowheads indicate representative living and dead cells, respectively. Scale bar, 50 μm. (C) For quantification of cell death, 300–400 cells were counted in four independent fields per coverslip. (D) Dose-dependent test of pro-apoptotic activity of CR-proBDNF. Cell death was assessed using DAPI staining 48 h after treatment with CR-proBDNF in LK medium. DAPI data were expressed as the percentage of mock cultures. In the cell death assay (C-D), n = 4 independent culture dishes. ANOVA followed by post-hoc analysis, *P < 0.05; **P < 0.01. Results were replicated in at least three independent experiments.

To test the biological effects of the CR-proBDNF *in vitro*, we purified the recombinant mutant protein using either the Baculovirus [22] or *E. coli *expression systems [23]. The identity of the purified proteins was confirmed using silver staining via analysis of their comigration with a molecular weight marker (~30-kDa) and detection on Western blot using a proBDNF-specific antibody (Fig. 3A). Baculoviruses expressed two BDNF isoforms that migrated at approximately 30- and 32-kDa; the latter was likely to be a glycosylated form (Fig. 3A, left). The CR-proBDNF protein expressed in *E. coli *formed inclusion bodies; therefore, we used urea denaturation for purification followed by refolding of the protein. The refolded CR-proBDNF exhibited a secondary structure, as assessed using circular dichroism (CD) spectroscopy [Additional file 2A].

To address the role of proBDNF on cell viability of CNS neurons, we first examined the function of *E. coli*-derived CR-proBDNF protein in cultured cerebellar granule neurons (CGNs), which express both p75^NTR ^and TrkB receptors [24]. CGNs require high levels of extracellular K^+ ^(HK, 26 mM) for survival. In addition, a low extracellular K^+ ^concentration (LK, 5 mM) induces apoptosis [25]. Cell viability of CGNs was assessed 48 h later using DAPI staining, which allows the visualization of apoptosis-induced chromatin condensation in the nucleus. Consistently with previous reports [26,27], matBDNF promoted the survival of CGNs, even when cell death was induced by LK (Fig. 3B, matBDNF, arrows). However, CR-proBDNF failed to prevent apoptosis induced by LK (Fig. 3B, CR-proBDNF, arrowheads). Quantitative analysis (Fig. 3C) revealed that 100 ng/ml CR-proBDNF promoted LK-induced apoptosis in CGNs and increased the number of dying cells by 57.0 ± 0.2% when compared with the mock culture (ANOVA followed by post-hoc analysis; **P < 0.01). In contrast, 100 ng/ml matBDNF significantly inhibited LK-induced apoptosis in CGNs when compared with the mock culture (39.0 ± 0.0%, **P < 0.01) [26,27]. These results suggest that CR-proBDNF promotes LK-induced apoptosis of cultured CGNs and that CR-proBDNF and matBDNF exert the opposite biological effect on the survival of CGNs.

A previous study demonstrated that recombinant cleavage-resistant proBDNF, at subnanomoler concentration, elicited apoptosis of NGF-deprived superior cervical ganglion (SCG) neurons [7]. We therefore investigated the effect of *E. coli*-derived CR-proBDNF on apoptosis of LK-treated CGNs at a similar concentration. A significant increase in the number of apoptotic CGN neurons was found even at 10 ng/ml (0.2 nM) CR-proBDNF, to a similar extent to that of 100 ng/ml (Fig. 3D). In addition, *E. coli*-derived CR-proBDNF failed to induce TrkB phosphorylation for up to 360 min after the application [Additional file 2B] and CR-proBDNF-induced cell death was not significantly found in CGNs derived from p75^NTR-/- ^mice [Additional file 2C, p75^NTR-/-^, *t*-test, *P *= 0.145, compared to Mock]. Finally, a proapoptotic effect was evident for CR-proBDNF derived from both Baculovirus and *E. coli*, whereas heat-denatured CR-proBDNF had no effect [Additional file 2D]. These results together suggest that *E. coli*-derived CR-proBDNF has a proBDNF biological activity to the similar to mammalian cell-derived proBDNF [7].

We also examined whether CR-proBDNF could affect the survival of CGNs cultured in HK medium, which promotes the survival of these cells [25]. Quantitative analysis of cell death using DAPI staining revealed that neither CR-proBDNF nor matBDNF had a significant effect on the number of CGNs in this survival-promoting medium [Additional file 2E], suggesting that proBDNF is proapoptotic in vulnerable neurons rather than in healthy neurons and that compared to peripheral neurons CNS neurons may not be so sensitive to proBDNF. Finally, it is notable that the BDNF polymorphisms, which lead to an inefficient cleavage of proBDNF, might be risk factors for defects in CGNs development and/or survival.

### CR-proBDNF inhibits neurite growth in basal forebrain cholinergic neurons

To examine whether proBDNF-induced apoptosis is a general phenomenon in all CNS neurons that express p75^NTR^, we chose to use basal forebrain cholinergic neurons (BFCNs), which are known to express high levels of p75^NTR ^[24]. A large body of evidence demonstrates that matBDNF promotes survival and differentiation of BFCNs [1]. A previous, yet initial, report characterized biological response of the BFCNs to matBDNF [28]: (1) the pro-survival effect was relatively more striking in low-density cultures, while the differentiated effects were more pronounced in high-density culture and (2) delayed application of matBDNF diminished the pro-survival effect even in low-density culture. In the present study, we sought to apply CR-proBDNF to rat BF neurons cultured in serum-containing medium for 2 weeks in order to see the biological action of proBDNF on BFCNs.

**Figure 4:**
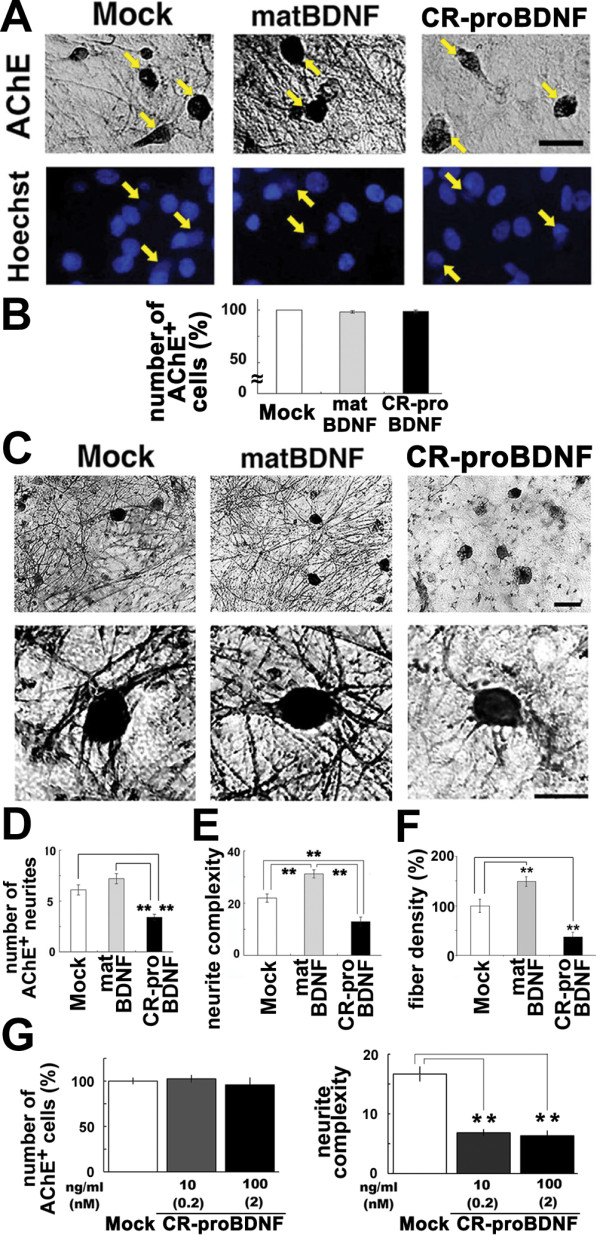
**Neurite growth of basal forebrain cholinergic neurons is inhibited by CR-proBDNF, but elicited by matBDNF**. BFCNs were cultured in serum-containig (A-E) or serum-free (F) medium for 2 weeks and treated with 100 ng/ml CR-proBDNF or matBDNF in the same medium condition for 2 days. Histochemistry and quantitation of AChE-positve neurites were done as described in Methods. (A) BFCNs were double-stained using AChE histochemistry and Hoechst 33258 (arrows). (B) The survival rate of BFCNs (%) = 100 × [living cells]/([living cells] + [dead cells]). Data were normalized to mock cultures (100% as control). n = 159 (Mock), 151 (matBDNF), and 149 (CR-proBDNF) from three independent coverslips. Results were replicated in three independent experiments. (C) Low- and high-magnification images of AChE-stained BFCNs. (D) The number of neurites extending outwards from the cell body shown in C. n = 38 (Mock), 37 (matBDNF), and 40 (CR-proBDNF) cells from three independent coverslips. (E) Neurite complexity as revealed by Sholl analysis. n = 30 (Mock), 30 (matBDNF), and 30 (CR-proBDNF) cells from three independent coverslips. In multi-bar graphs, ANOVA followed by post-hoc analysis was used. **P < 0.01. Results were replicated in at least three independent experiments. (F) The opposing effects of matBDNF and CR-proBDNF on neurite fiber density were confirmed by a distinct quantitative method. The maximal threshold of AChE-positive cholinergic fiber intensity was defined as 70% above the background. The total intensity of the fibers was determined in an optical field and was divided by the number of AChE-positive neurons in the same field. Data were collected from four independent fields in a single chamber. *t*-test, **P < 0.01, compared to Mock (100% as control); n = 6 independent culture dishes. Scale bar, 5 μm (A and C). (G) Effect of CR-proBDNF on neurite density of BFCNs in serum-free condition and a dose-dependency test of CR-proBDNF. Cell survival (left) and the neurite number of BFCNs (right) were determined. Note that proBDNF negatively regulates the neurite density of BFCNs in serum-free conditions and at subnanomoler concentration. n = 3 independent coverslips. *t*-test, **P < 0.01, significantly different from Mock.

Consistently with previous reports [28,29], 4-day treatment with matBDNF (100 ng/ml) markedly increased the number and complexity of acetylcholine esterase (AChE)-positive neurites of the 2-week cultured BFCNs (Fig. 4A and 4C, matBDNF). The cell viability of the AChE-positive BFCNs was assessed by DAPI staining (Fig. 4A). Unlike what was observed in CGNs, neither matBDNF nor CR-proBDNF (*E. Coli*-derived recombinant protein) has a significant effect on the number of the survived BFCNs and on the nuclear morphology (Fig. 4A, arrows, and 4B). However, CR-proBDNF caused the opposite effect on fiber density in BFCNs: treatment with 100 ng/ml CR-proBDNF for 4 days led to a marked reduction in the neurite number of AChE-positive neurites extending outwards from the cells (Fig. 4D, CR-proBDNF), without affecting the survival of these cells (Fig. 4C, CR-proBDNF). Quantitative analysis showed that CR-proBDNF decreased the number of primary fibers by more than 45% (Fig. 4D, CR-proBDNF). In contrast, matBDNF increased the number of the AChE-positive fibers by approximately 17% (Fig. 4D, mBDNF). We performed several methods to evaluate of neurite growth. Sholl analysis, which is used to assess the number and complexity of fibers extending out from the cell body [30], revealed that CR-proBDNF significantly reduced the number of cholinergic fibers in the first 10 μm from the cell body by 42% (Fig. 4E, CR-proBDNF). We also determined the neurite density of BFCNs in culture dishes (intensity of cholinergic fibers/number of cholinergic neurons). The opposing effects of matBDNF and CR-proBDNF on the fiber density were confirmed by this quantitative method (see Methods): Mock 100 ± 13.6% (as control); matBDNF, 149 ± 9.3%**; CR-proBDNF, 37.3 ± 9.4%** (*t*-test, **P < 0.01, compared to Mock; n = 6 independent culture dishes) (Fig. 4F). These results together suggest that CR-proBDNF inhibits neurite growth of BFCNs while matBDNF enhances this growth.

Finally, we examined whether CR-proBDNF had the similar effect on fiber density of BFCNs in serum-free condition. To this end, we cultured BFCNs in serum-free medium containing 5 μg/ml transferrin, 5 μg/ml insulin, 20 nM progesterone and 10 μM cytosine arabinoside (AraC) [29] for 2 weeks and applied CR-proBDNF to the neurons in the same medium. Four-day treatment with CR-proBDNF significantly decreased the fiber density, but not cell viability, of BFCNs, and even at subnanomoler concentration the effect of CR-proBDNF was significant (*t*-test, **P < 0.01) compared to that of un-treated cultures (Fig. 4G). These data suggest that proBDNF acts to BFCNs in a direct manner for negative regulation of the neurite density.

### CR-proBDNF reduces dendritic spine density of hippocampal neurons in primary and slice cultures

**Figure 5:**
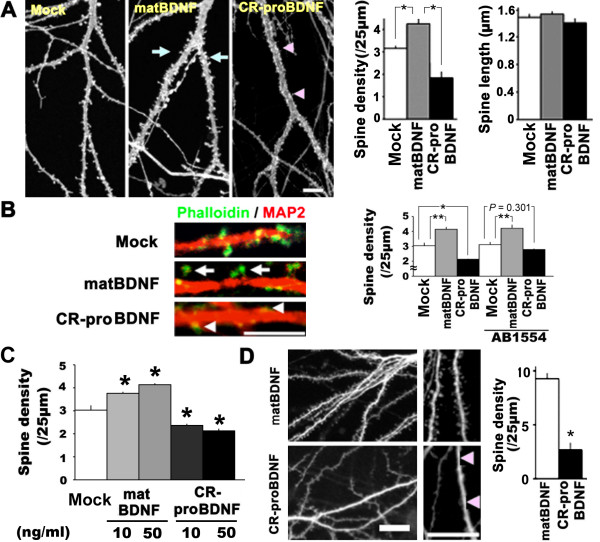
**proBDNF reduces dendritic spine density in hippocampal neurons**. Cultured neurons were maintained for 3–4 weeks and treated with the indicated reagents (50 ng/ml) for 2 (A, B, and D) or 3 days (C). In A and B, neurons were cultured in serum-containing medium. (A) CR-proBDNF reduced the density of DiI-labeled spines. Representative images of DiI-labeled neurons treated with the indicated reagents for 2 days (left). Summary of spine density (middle) and length (right). Data were collected from 52 (Mock), 59 (matBDNF), and 38 (CR-proBDNF) independent cells (non-parametric test, *P < 0.05, compared to Mock). Note that matBDNF increased spine density, while proBDNF reduced spine density (arrows and arrowheads). (B) proBDNF promoted the shrinkage of phalloidin-labeled spines and p75^NTR ^was involved in the proBDNF action. Application of a functionally blocking antibody against p75^NTR^, AB1554 was performed and fixed neurons were stained with an anti-MAP2 antibody and FITC-labeled phalloidin. Representative images of the double-stained neurons (left) and a quantitative analysis of phalloidin-labeled spiny structures (right) are shown, n = 30–40 independent cells from three independent coverslips. *t*-test, *P < 0.05, **P < 0.01, compared to Mock (100% as control). (C) CR-proBDNF decreases spine density in low-density hippocampal neurons cultured in serum-free medium. The cultures were incubated with CR-proBDNF in serum-free medium for 2 days and double-stained with an anti-MAP2 antibody and FITC-labeled phalloidin. n = 24–32 cells from three independent coverslips. *t*-test, *P < 0.05, **P < 0.01, compared to Mock. (D) CR-proBDNF decreased spine density in hippocampal slices. Representative images of DiI-labeled neurons at low and high magnification are shown (left). A decrease in spine density was observed in CR-proBDNF-treated slices (arrow heads). Quantification of spine density (right). Data were collected from 7 (Mock) and 6 (CR-proBDNF) independent slices. Note that CR-proBDNF-treated slices showed a decrease in spine density when compared with matBDNF. *t*-test, *P < 0.05. Scale bar, 10 μm (A, B, and D).

As proBDNF facilitates LTD [15], proBDNF may have distinct biological roles in synaptic plasticity [10]. To test this possibility, we cultured hippocampal neurons (6–8 × 10^4 ^cells/cm^2^) for 3–4 weeks and then applied 50 ng/ml of matBDNF or proBDNF for 2 days. We first performed DiI labeling of dendritic spine structures [31]. Consistently with a previous report [32], matBDNF dramatically increased spine density in hippocampal neurons (Fig. 5A, matBDNF, arrows). However, CR-proBDNF appeared to promote the density of spine protrusions (Fig. 5A, CR-proBDNF, arrowheads), which suggests that proBDNF negatively regulates dendritic spine density in CNS neurons. To evaluate this effect of CR-proBDNF in a quantitative manner, we assessed the density of dendritic spines with a typical mushroom head and length (> 1 μm) on the proximal region of dendrites, as described [31,33]. Quantitative analysis demonstrated that matBDNF increased dendritic spine density by 34.9 ± 8.9%, whereas CR-proBDNF decreased spine density by 41.6 ± 9.0% (Fig. 5A, left graph, nonparametric test; *P < 0.05 when compared with Mock). Finally, cultured hippocampal neurons treated with heat-denatured CR-proBDNF (50 ng/ml, 2 days) had a dendritic spine density that was 77.2 ± 0.1% higher than that observed with CR-proBDNF (*t*-test, **P < 0.001, compared to CR-proBDNF-treated cells, n = 41 (CR-proBDNF), 77 (heat-denatured CR-proBDNF) independent cells), suggesting that the reduction of dendritic spine density is a biological activity of proBDNF protein.

We further visualized spine protrusions using the F-actin-binding dye phalloidin [34]. Similarly to the results obtained in Fig. 5A, matBDNF and CR-proBDNF had opposite effects on dendritic spine formation (Fig. 5B, arrows and arrowheads). Quantitative analysis in a similar manner to Figure 5A the similar CR-proBDNF decreased the density of phalloidin-labeled spines by 41.6 ± 9.0% (Fig. 5B, right, CR-proBDNF, nonparametric test; *P < 0.05 when compared with Mock). We further tested whether a functionally blocking antibody against p75^NTR^, AB1554 [35,36] inhibited the effect of CR-proBDNF on spine density, because it was reported that, after 14 d *in vitro*, hippocampal neurons allowed the significant colocalization of p75^NTR ^with PSD95 [15]. Treatment with AB1554 (1/100 dilution) significantly rescued the effect of CR-proBDNF on spine density (Fig. 5B, right, CR-proBDNF + AB1554), suggesting that proBDNF involves p75^NTR ^on the neuronal surface to reduce the spine density. We next examined the effect of CR-proBDNF (50 ng/ml, 2 days) on the number of MAP-2-positive neurons in culture. Quantitative analysis revealed that CR-proBDNF did not affect the survival nor the overall dendritic morphology of the cultured mature hippocampal neurons used in this experiment [Additional file 3], suggesting that this effect of CR-proBDNF on spine density may not be due to the proapoptotic effect of proBDNF.

Additionally, we examined whether CR-proBDNF affected spine density in serum-free and low-density cultures of hippocampal neurons, as described in Methods. In this culture, the percentage of contaminated glial cells was 7.0 ± 1.4% (n = 4 independent culture dishes). Treatment of CR-proBDNF for 2 days led to a significant decrease in spine density and 10 ng/ml (0.2 nM) was sufficient for the negative action of CR-proBDNF (Fig. 5C). These data support the notion that proBDNF reduces spine density of hippocampal neurons in a direct manner.

**Figure 6:**
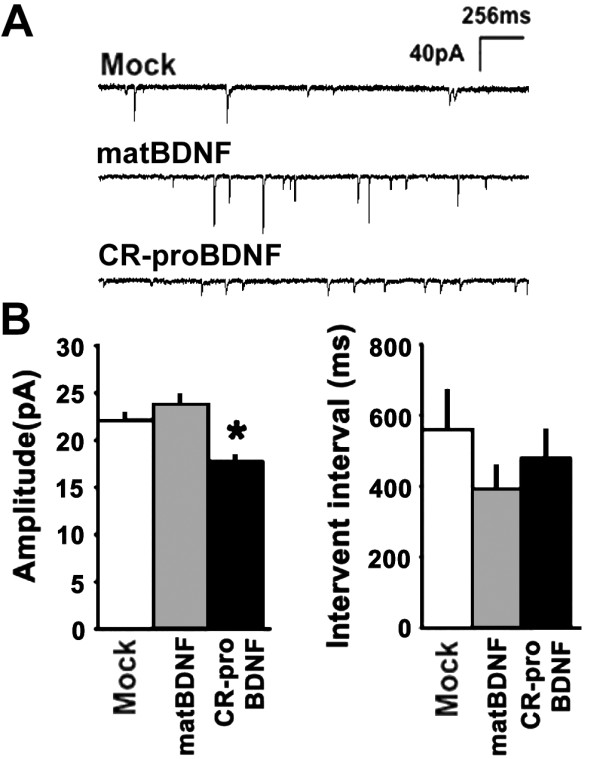
**CR-proBDNF attenuates amplitude of functional synaptic transmission in cultured hippocampal neurons**. Cells were cultured as described in Fig. 5A and treated with the indicated reagents for 3 days. A patch-clamp technique in the whole-cell configuration was used to record mEPSCs from pyramidal-shaped neurons and all data were statistically evaluated. (A) Representative mEPSCs. (B) Amplitude (left) and frequency (right) of mEPSCs. Note that proBDNF significantly decreased the amplitude, but not the frequency, of mEPSCs. Data were collected from 27–31 independent cells (non-parametric test, *P < 0.05, significantly different from Mock).

Finally, to evaluate this novel effect of CR-proBDNF in physiological contexts, we carried out two additional experiments. First, we investigated the effect of CR-proBDNF on dendritic spine density in hippocampal slice cultures. Interestingly, CR-proBDNF (50 ng/ml, 2 days) reduced the density of spines in hippocampal slice cultures (Fig. 5D, CR-proBDNF). Second, we recorded spontaneous miniature excitatory postsynaptic currents (mEPSCs) [37,38] in cultured hippocampal neurons (21 days *in vitro*) after 2-day treatment with matBDNF or CR-proBDNF (50 ng/ml). Hippocampal neurons treated with CR-proBDNF exhibited basal synaptic transmission (Fig. 6A), which support the notion that CR-proBDNF does not affect the survival of more than 3 week-cultured hippocampal neurons [Additional file 3]. However, proBDNF treatment significantly decreased the amplitude of mEPSCs (Fig. 6B, left, nonparametric test, *P < 0.05 when compared with the Mock group). As reported previously [39], the amplitude of mEPSCs did not increase significantly after matBDNF treatment. Neither matBDNF nor CR-proBDNF significantly affected the frequency of mEPSCs (Fig. 6B, right). These results together suggest that proBDNF reduces dendritic spine density of CNS neurons in slice culture and attenuates the amplitude of functional synaptic transmission.

## Discussion

Since the discovery that proNGF binds p75^NTR ^with a higher affinity than matNGF and that it induces apoptosis rather than enhancing cell survival in sympathetic neurons [4], proneurotrophin-p75^NTR ^signaling and function have returned to the center stage in the field of neurotrophin research. There is a pressing need to understand the cleavage of proneurotrophins, as well as their biological functions in different brain regions. A crucial, yet poorly solved, issue is whether proBDNF has a physiological function [13]. In the present study, based on a predictive study of BDNF protein variants, we first provided molecular and cellular insights on the possible consequences that the rare human SNPs have near the cleavage site of proBDNF (Figs. 1 and 2). Second, we demonstrated that the poorly cleaved proBDNF (CR-proBDNF) enhances apoptosis in cultured CGNs (Fig. 3). Finally, we demonstrated the novel actions of proBDNF, which include the inhibition of neurite outgrowth in BFCNs (Fig. 4) and the reduction of dendritic spine density in mature hippocampal neurons (Fig. 5). These results suggest that proBDNF cleavage is a crucial step for the negative regulation of neurotrophic actions in the brain and that proBDNF exerts multiple biological actions on CNS neurons.

Although the rare human BDNF polymorphisms studied here are validated by the National Center for Biotechnology Information (NCBI) and the mutations cause amino acid substitutions near the cleavage site of proBDNF, the functional and physiological impacts remain ill-defined. Applying a bioinformatic analysis (PONDR), we predicted that the mutants would undergo disordered-to-ordered transitions around the proteinase cleavage site of proBDNF (Fig. 1B). The inhibition of proteolytic cleavage of the BDNF polymorphic variants was demonstrated by Western blot analysis of cultured neurons transfected with constructs encoding the variant forms of proBDNF (Fig. 1C) and an in vitro protease digestion assay [Additional file 4]. Interestingly, it was reported that a specific plasmin-cleavage could occur after Arg125 rather than Arg128 of the RVRR sequence [40]. Nakayama (1997) also described in a review article that since furin cleavage is essential for the production of a wide variety of biologically active proteins it is possible that mutation of the furin cleavage site of the precursors may result in genetic disorders [41]. Additionally, we have recently generated a knock-in mouse line by replacing the endogenous BDNF allele with a CR-proBDNF-encoding construct and found inefficient processing of the mutant proBDNF protein (data not shown), suggesting that the human BDNF polymorphisms would cause reduced the conversion of proBDNF into matBDNF *in vivo*.

We further demonstrated that the intracellular distribution (trans-Golgi and secretory granules) and subcellular localization (dendrites and soma) of CR-proBDNF were normal (Fig. 2), which suggests that the amino acid substitutions *per se *do not alter intracellular sorting and trafficking and supports the notion that endogenous proBDNF is transported [12] and secreted in an activity-dependent manner [42]. In this context, these findings are in marked contrast to those obtained for the Val66Met variation of BDNF, which leads to the striking alteration of the sorting of BDNF to the secretory granules and of its subsequent activity-dependent secretion [18,43].

Two recent reports described the mechanisms of BDNF secretion [11,12]. In mixed cultures of neurons and glial cells, proBDNF was rapidly converted into matBDNF and was secreted as matBDNF. In neuron-rich cultures, however, proBDNF was the predominant form released. These two reports suggest that proBDNF cleavage in the nervous system is regulated in a more specific manner and on cellular context [13]. Although our results stemmed from overexpression experiments, the inhibition of proBDNF cleavage significantly reduced the content of BDNF in cells and resulted in the predominant secretion of the proBDNF when compared with wild-type BDNF. Our in silico prediction study suggested that the prodomain was structurally disordered thus rendering it likely to be degradation (Fig. 1B). Together, these results raise questions about the roles of the BDNF prodomain and the stable presence of proBDNF inside and outside of neurons.

Based on studies on proNGF, the concept is emerging that mature neurotrophins promote cell survival, while proneurotrophins facilitate apoptosis [9]. A recent study showed that proBDNF also promotes the death of sympathetic neurons, which extends the pro-apoptotic role to a second member of the neurotrophin family [7]; however, little is known about the inhibitory action of proBDNF on the survival of CNS neurons [8]. Using primary cultures of CGNs derived from rat and p75^NTR^-knockout mice, we demonstrate that CR-proBDNF enhances LK-induced apoptosis in CGNs (Fig. 3, [Additional file 2]). It is also noteworthy that we have developed a simple protocol to generate recombinant proBDNF from *E. coli*. (Fig. 3A, [Additional file 2A]). First, the recombinant proBDNF, which, even at subnanomolar concentrations, significantly promoted apoptosis of cultured CGNs (Fig. 3D), [Additional file 2]. Second, *E. coli*-derived CR-proBDNF failed to induce TrkB phosphorylation for up to 360 min after the application [Additional file 2B]. Third, similar to the previous reports [7,8], in the absence of p75NTR, E. coli-derived CR-proBDNF failed to promote apoptosis of CGNs [Additional file. 2B]. We also showed that silkworm-derived CR-proBDNF is capable of promoting apoptosis of cultured CGNs. These results are conceptually parallel to previous reports using recombinant proBDNF derived from mammalian cells [7,8].

The extent of the opposite actions of proBDNF and matBDNF in the CNS has yet to be determined extensively. Intriguingly, the present study demonstrated that proBDNF is capable of reducing the extent of neurite branching in BFCNs (Fig. 4), which are a well-known population of p75^NTR^-highly expressing neurons in the adult brain [24]. However, the morphological action is inconsistent with that of a previous report demonstrating that BF neurons exhibited apoptosis in response to proNGF and proBDNF in culture [8]. One explanation for such a discrepancy is that there were likely differences in maturation of the neurons in culture dishes. We applied CR-proBDNF to BFCNs cultured for more than 2 weeks. A previous report demonstrated that delayed application of matBDNF promoted neuronal differentiation rather than cell survival of the neurons [28]. Since this effect of CR-proBDNF was observed both in serum-containing and serum-free medium (Fig. 4G), we have reasoned that proBDNF may act to the BFCNs in a direct manner. Another interesting finding was that proBDNF reduced dendritic spine density in mature hippocampal neurons: hippocampal neurons treated with CR-proBDNF exhibited a marked shrinkage of spines, both in dissociated culture and in slice culture. This new finding leads us to formulate several important suggestions. First, matBDNF and proBDNF may play opposite roles in structural plasticity: matBDNF and proBDNF may contribute to the augmentation and elimination of synaptic connections, respectively (Fig. 5). Second, it was recently demonstrated that low-frequency stimulation induced predominantly the secretion of endogenous proBDNF from cultured hippocampal neurons [42]. Short-term exposure of CA1 synapses to proBDNF facilitates LTD [15]. We showed that CR-proBDNF, which decreased the density of dendritic spines in hippocampal neurons, also reduced the amplitude of mEPSCs (Fig. 6). In addition to these studies using dissociated hippocampal neurons on the feeder layer of glial cells, proBDNF decreased spine density in a low-density culture and the effect was significant even at subnanomolar concentration (Fig. 5C). The treatment with CR-proBDNF did not affect the number of MAP2-positive neurons in the culture dishes [Additional file 3]. These additional data support the notion that proBDNF acts on spine structures in a direct manner.

However, the mechanism underlying proBDNF-mediated induction of dendritic spine shrinkage remains unclear. Immunohistochemical study demonstrated that, after 14 d in vitro, p75NTR immunoreactivity as punctate staining around the cell body as well as dendrites, with distribution very similar to that of PSD95 immunoreactivity [15]. Zagrebelsky *et al*. reported a crucial role of p75^NTR ^on spine formation by gain-of function and loss-of function studies [44]. In the present study, the inhibitory action of CR-proBDNF on spine density was rescued by a p75^NTR ^functional blocking antibody (Fig. 5B), suggesting that proBDNF/p75^NTR ^signaling reduces spine density of hippocampal neurons. It was reported that the transport of the signaling complex that contains the p75^NTR ^receptor and activated Rap1 plays a role in the effect of myelin-associated glycoprotein (MAG), which is a well-characterized axon growth inhibitor [45]. During LTD, Rap mediates the NMDA-R-dependent removal of synaptic AMPA receptors [46]. Furthermore, LTD induces the shrinkage of dendritic spines via the action of cofilin [47]. We also found that the CR-proBDNF-dependent morphological change of dendritic spines in cultured hippocampal neurons appeared in 30 min (data not shown). These recent reports, together with our findings, may provide several clues for the elucidation of the molecular mechanism underlying the proBDNF-dependent synaptic depression.

## Conclusion

The present study suggested that the function of proBDNF in the CNS is not limited to apoptosis and consolidation of LTD [10], and that in a cell type-specific fashion proBDNF affects dendritic growth, spine growth, and cell survival of CNS neurons. Very recently, it has been demonstrated that proBDNF eliminated presynaptic terminals using Xenopus nerve-muscle co-cultures [48]. Hence proBDNF itself and the proteolytic cleavage of proBDNF could play important roles in the regulation of BDNF signaling in the architecture of the nervous system.

## Methods

### Materials

Recombinant human matBDNF was kindly provided by Sumitomo Pharmaceuticals (Osaka, Japan). NGF was obtained from mouse submaxillary gland [27]. Recombinant human NT-3 protein is from Sigma (St. Louis, MO). The anti-SgII (secretogranin II) antibody was kindly provided by Dr. T. Watanabe (Asahikawa Medical College, Asahikawa, Japan). The anti-BDNF (N-20) and anti-c-Myc (9E10) were from Santa Cruz Biotechnology (Santa Cruz, CA); the anti-GFP antibody was from MBL (Nagoya, Japan); the anti-phospho-Trk (Y490) was from Cell Signaling (Beverly, MA); anti-MAP2 (microtubule-associated protein 2) and anti-GFAP (glial fibrillary acidic protein) antibodies were from Sigma; the anti-TGN38 (trans-Golgi network 38) and anti-TrkB (TrkB_out_-specific antibody: clone 47) antibodies were from BD Biosciences (Dickinson, San Jose, CA); the Alexa Fluor-conjugated secondary antibodies were from Molecular Probes (Eugene, OR). p75^NTR ^functional blocking antibody (AB1556) was from Chemicon (Temecula, CA).

Hoechst 33258 (bisbenzimide), glutamate, and phalloidin (phalloidin-FITC) were from Sigma; 4',6'-diamidino-2-phenylindole dihydrochloride (DAPI) and 1,1'-dioctadecyl-3,3,3',3'-tetramethylindocarbocyanine perchlorate (DiI) were from Molecular probes; lactate dehydrogenase (LDH) cytotoxicity test was from Wako (Tokyo, Japan). Transferrin, insulin, progesterone, cytosine arabinoside (AraC) and polyethylenimine were purchased from Sigma.

Wistar ST rats and C57BL/6 mice were from NIPPON SLC (Hamamatsu, Japan). B6C3F1 mice were from the Charles River Laboratory (Kanagawa, Japan). The p75^NTR ^knockout mice (ngfr^tm1Jae^) were from The Jackson Laboratory (Bar Harbor, ME). All animal experiments were strictly in accord with the protocols approved by the Institutional Animal Care and Use Committee of the AIST.

### Primary cultures of CNS neurons and application of the indicated reagents

Primary cultures of dissociated rat and mouse CGNs were prepared from the cerebella of P9 rats and P6-7 p75NTR^-/- ^mice, respectively, according to a previous report [27] with some modifications. Briefly, cells were dissociated using a plastic pipette after digestion with papain (90 units/ml, Worthington, Lakewood, NJ) at 37°C and were then cultured in minimum essential medium (MEM, Gibco, Carlsbad, CA) that contained 5% fetal bovine serum (FBS), 5% horse serum, to a final cell density of 5 × 10^5 ^cells/cm^2 ^on polyethyleneimine-coated culture plates. After culture for 1 day in a humidified CO_2 _(5%) incubator, the medium was changed to Minimum Essential Medium (MEM) that contained 26 mM potassium (HK) and was supplemented with 5% HS and 20 μM AraC. After 4 days in culture, cells were placed in serum-free MEM containing 5.4 mM potassium (LK) or HK-MEM, for experimentation. In separate cultures of CGNs, the percentage of GFAP-positive glial cells was 4.3 ± 0.72% (n = 4 independent culture dishes).

Dissociated BFCN cultures were prepared using E20 rats (Wistar ST) as described [49,50], with a few modifications. Briefly, the basal forebrain area containing the septal nuclei was dissected and digested by papain/DNaseI. Dissociated neurons were seeded onto polyethylenimine-coated 8-well chamber slide (Nunc, Rochester, NY). To maintain the cell survival, serum-containing medium composed of 5% FBS, 5% heat-inactivated HS, and 90% Dulbecco's minimum essential medium (DMEM, Invitrogen, Carlsbad, CA) was used and cells were cultured to a final density of 3 × 10^5^cells/cm^2 ^on polyethyleneimine-coated culture plates. The medium was changed every 7 days. After 14 days in culture, the cells were treated with the indicated reagents in serum-containing medium, described above. In this preparation, GFAP-positive glial cells formed a feeder layer under the neurons (data not shown). In separate experiments (Fig. 4G), BFCNs were maintained and treated with the indicated drugs in serum-free defined medium containing 5 μg/ml transferrin, 5 μg/ml insulin, 20 nM progesterone and 20 μM AraC [29]. The medium was changed every 3 days. Then, in sister cultures, the percentage of GFAP-positive glial cells was calculated to be 11.4 ± 2.1% (n = 3 independent culture dishes).

For quantitative analysis of dendritic spine density, dissociated hippocampal neurons were prepared according to previous reports [37,51] with some modifications. For the experiments of Figs. 5A and 5B, hippocampi were dissected from embryonic day 18–20 rats and treated with 0.125% trypsin for 15 min at room temperature. The dissociated neurons were plated on polyethylenimine (0.01%)-coated glass coverslips (Matsunami, Osaka, Japan) at a density of 6–8 × 10^4 ^cells/cm^2^. The neurons were first cultured in DMEM containing 5% FBS and 5% HS. After 4 d in culture, AraC (2 μM) was added for 24 h to inhibit proliferation of non-neuronal cells. The neurons were then maintained in DMEM containing 10% HS for up to 3 weeks. Half of the medium was changed twice weekly. GFAP-positive glial cells formed a feeder layer under the neurons (data not shown). In separate experiments (Fig. 5C), dissociated neurons (2 × 10^4 ^cells/cm^2^) were plated on polyethylenimine (0.01%)-coated plastic Petri-dishes (Corning Costar, Corning, NY) and co-cultured with confluent glial cells cultured on insert cup but separated by a microporous membrane filter and co-cultured in a common medium (B27/Neurobasal medium, Invitrogen) supplemented with 10 μM AraC. The medium was changed every 3 days. The biological assay of Fig. 5C was performed for 3–4 week cultured neurons. For immunoprecipitation (Fig. 1F), primary cultures of cortical neurons (3 × 10^5 ^cells/cm^2^) were prepared from E20 rats [52]. Three-day cultured cerebral cortical neurons were infected with wild-type or mutant BDNF-expressing Sindbis viruses for 12 h and maintained in serum-containing medium for 3 days. Supernatants were then collected for the following immunoprecipitation experiment.

### Bioinformatical prediction of proBDNF structure

Predictor Of Naturally Disordered Regions (PONDR) algorithm (, Molecular kinetics, Indianapolis, IN) was carried out according to the report of Romero *et al*. [16,53]. The PONDR-based prediction of unstructured regions was performed using the VL-XT predictor, which stipulates that a residue value that exceeds a threshold of 0.5 pinpoints a disordered region.

### DNA constructs and Sindbis virus infection

The Sindbis vector pSinEGdsp was generously provided by Dr. H. Nawa (Niigata University, Niigata, Japan). In this plasmid, two subgenomic promoters were arranged in tandem and the recombinant enhanced GFP (EGFP) gene was subcloned downstream of the second subgenomic promoter [19]. The human BDNF cDNA was subcloned into the pBluescript vector (Stratagene, La Jolla, CA). The Myc epitope tag was added to the 3' end of BDNF cDNA using a PCR method and the resulting BDNF-Myc fragment was subcloned into the pCR-Blunt II Topo Vector (Invitrogen). The variants with substituted Arg to Met at codon 125 and Arg to Leu at codon 127, as well as the double substitution (Arg125Met and Arg127Leu), were generated using the PCR-based Site Directed Mutagenesis Kit (Stratagene). The integrity of all constructs was confirmed by sequencing. For the expression of the EGFP fusion protein, each DNA fragment was subcloned into the EGFP-N1 vector (Clontech, Palo Alto, CA) [18]. The BDNF-Myc constructs and the BDNF-GFP constructs were subcloned into the Sindbis vectors pSinRep5 (Invitrogen) and pSinEGdsp, respectively. Sindbis viruses were produced according to the manufacturer's manual (Invitrogen). Sindbis virus infection was performed as described [18]. The efficiency of the infection was monitored by GFP fluorescence and the titer used was sufficient for approximately 100% transduction efficiency for Western blotting and 10% for immunostaining, respectively.

### Recombinant CR-proBDNF produced by the Baculovirus expression system

The human cDNA CR-proBDNF (R125M/R127L-BDNF) was inserted into the Baculovirus transfer vector pYNGproBDNF_ML_, which was cotransfected with BmNPV (CPd strain) DNA into the Bm cell line (BmN) (Katakura Industries, Saitama, Japan) [22]. Recombinant viruses were screened using the endpoint dilution method and were injected into the body cavities of fifth-instar silkworm larvae (2 × 10^6 ^p.f.u./head). The recovered haemolymph samples were centrifuged at 12,000 × g for 60 min and the supernatants were stored at -80°C. The mixture was diluted in Tris solution (10 mM Tris, 10 mM Bis-Tris Propane, 200 mM NaCl, pH 6.0) and was then loaded onto an anion exchange column (Poros HQ/1-2312-26, Applied Biosystems, Foster, CA). After washing the column with Tris solution, the recombinant proBDNF was eluted using a linear gradient of NaCl (200 to 1000 mM). Recombinant proteins were dialyzed in the Tris solution and stored at 4°C until further use. Purification was monitored by Western blot analysis using an anti-proBDNF antibody as well as silver staining (Fig. 3).

### Recombinant CR-proBDNF produced by the E. coli expression system

The His tag was added to CR-proBDNF (R125M/R127L-BDNF) by PCR; the resulting construct was subcloned into the pET-19b vector (Novagen, Madison, WI) and was bidirectionally sequenced. Approximately 6 g of *E. Coli *inclusion bodies (IBs)-containing inactive and aggregated recombinant His-tagged proBDNF_ML _were solubilized with BugBuster (Novagen). After centrifugation (17,000 rpm, 20 min, 4°C), the IBs were resuspended in BugBuster. This step was repeated 2–3 times. The IBs were solubilized with 8 M urea solution (8 M urea, 0.5 M Tris-HCl, pH 8.5, 1 mM EDTA, 40 mM DTT) at 4°C overnight. Derivatization was achieved by adding N- [Tris(hydroxymethyl)methyl]-3-amino propanesulfonic acid (TAPS)-sulphonate (final concentration, 125 mM; Wako) followed by incubation at 4°C overnight. His-tagged CR-proBDNF was purified using Ni-bead chromatography (Invitrogen), according to the manufacturer's instructions and using a 150 mM imidazole solution for elution. For the refolding of denatured His-tagged CR-proBDNF, dialysis (in 2 M urea, 0.2 mM oxidized cysteamine, 5% glycerol, 50 mM Tris-HCl, pH 7.4) was conducted at 4°C overnight [23]. For biological assays, the resultant protein was dialyzed against phosphate-buffered saline (PBS) and was stored at 4°C until further use. Purification was monitored by Western blot analysis using anti-proBDNF antibodies as well as silver staining (Fig. 3).

### Generation of proBDNF-specific antibodies

The entire human prodomain sequence (aa 19–126) of BDNF was expressed in Rosettagami-2 bacterial cells as a C-terminal fusion to GST using the pGEX4T-3 vector. The GST fusion protein was purified using the GST-bind kit, according to the manufacturer's protocol (Novagen), and was used to immunize mice and to screen hybridoma clones. Clone mAb287 was selected from the initial screen and was further characterized using recombinant proteins and cell and brain lysates. A polyclonal anti-proBDNF antibody was raised in rabbits against the recombinant prodomain (aa 19–126) of the human BDNF (which was generated in *E. coli*) conjugated to keyhole limpet hemocyanin (KLH) for immunization. The antibody was affinity-purified against the antigenic protein using the AminoLink Kit (Pierce Biotechnology, Rockford, IL). A chick polyclonal anti-proBDNF antibody was raised against the KLH-conjugated peptide fragment NNKDADLYTSRVMLSSQ, which corresponds to aa 83–99 of the prodomain of human BDNF. The antibody was affinity-purified against the antigenic peptide (Aves Labs, Tigard, OR).

### Western blot analyses

The lysates of cultured neurons were prepared as described [52]. Briefly, cultured cells were rinsed three times with ice-cold PBS and were quickly lysed in cold lysis buffer containing 50 mM Tris-HCl (pH 7.4), 1 mM EDTA, 150 mM NaCl, 10 mM NaF, 1 mM Na_3_VO_4_, 1% Triton X-100, 10 mM Na_2_P_2_O_7_, 100 μM phenylarsine oxide, and 1% protease inhibitor cocktail (Complete Mini, Roche Diagnostics, Hertforshire, UK). Lysates were boiled for 5 min at 100°C and were then sonicated. The protein concentration of the supernatants was determined using the bovine serum albumine (BSA) standards from Pierce Biotechnology. The lysates were resolved by SDS-polyacrylamide gel electrophoresis (SDS-PAGE). Western blot protocols were according to Suzuki *et al*. (2004) [52] but modified for detecting signals more specifically. Samples were sonicated in an equal volume of SDS sample buffer (0.125 M Tris-HCl, pH 6.8, 20% glycerol, 4% SDS, and 10% 2-mercaptoethanol), heated at 100°C for 3 min, and resolved by SDS-PAGE. Samples were electrotransferred to polyvinylidene fluoride membranes (Immobilon P membrane, Millipore, Bedford, MA), which were blocked in Tris-buffered saline (TBS) containing 0.2% Tween-20 (TBS-T) and 3–5% BSA or Block Ace (Dainippon pharmaceutical, Osaka, Japan) and were then incubated with the indicated primary antibodies in TBS-T containing 0.3–0.5% BSA (TBS-TB) or 1–10% Block Ace at room temperature for 60–90 min. After washing 3 times with TBS-T, membranes were incubated with peroxidase-conjugated secondary antibodies in TBS-TB at room temperature for 30 min, and then washed 3 times with TBS-T. The chemical luminescence signals were detected using the ImmunoStar Reagents (Wako) or the SuperSignal West Femto Maximum Sensitivity Substrate (Pierce Biotechnology). The exposure time was adjusted so that the intensities of the bands were within the linear range. For quantification of the relative amounts of proBDNF and matBDNF, the blots were scanned and the images were converted to Tiff files and quantified using the NIH Image software (ImageJ version 1.37v). The relative amounts of proBDNF and matBDNF were obtained by normalizing the values to total BDNF values. Signals for proBDNF and matBDNF in a given lane of the blot (after subtraction of the background in the same lane) were added to obtain the value for total BDNF and were normalized to the signal of the GFP band. The data were averaged and were presented as mean ± standard error of the mean (SEM).

### Immunoprecipitation

Protein G Sepharose (15 μl gel, Amersham Pharmacia, Piscataway, NJ) was added to the conditioned culture medium of the dissociated cerebral cortical neurons transduced with Myc-tagged CR-proBDNF (R125M/R127L-BDNF) constructs and the mixture was rotated at 4°C for 60 min. Anti-c-Myc monoclonal antibody-conjugated agarose beads (20 μl gel, BD, San Jose, CA) were added to the supernatant after removal of the Protein G Sepharose. Incubation of the lysates with the beads was performed at 4°C overnight. The immune complexes were pelleted and immunoblotted using antibodies against BDNF or proBDNF.

### Cell viability assay

Cell viability was quantified by counting the number of dead cells using DAPI or Hoechst staining [54], and by measuring LDH released into the medium using the kit (Wako) [27]. For the LDH assay, culture media were centrifuged at 10,000 g for 10 min and 50 μl of dye buffer were added to 10–20 μl of each supernatant. The mixtures were incubated at 37°C for 15 min and 50 μl of terminating buffer was added to each sample. The absorbance was photometrically determined at a wavelength of 560 nm. For DAPI and Hoechst staining, cells were fixed using 4% paraformaldehyde/PBS for 20 min and were then stained with 100 ng/ml DAPI or 1 μg/ml Hoechst 33258 in PBS for 15 min. The ratio of condensed to intact nuclei was quantified based on observations using a fluorescence microscope.

### AChE histochemistry and quantitative analysis

Fixation and immunohistochemistry of cultured cholinergic neurons were performed according to previously described methods [49,50]. AChE histochemistry was performed as described [55]. The phase-contrast images of AChE-positive cholinergic fibers and the cell body with a diameter of > 2.5 μm were acquired using a cooled CCD camera (MRc5, Carl Zeiss, Swiss) mounted on a Nikon microscope (TE230, Nikon, Japan). Sholl analysis was performed to determine the number of AChE-positive neurites at a distance of 10 μm from the soma [30]. The number of AChE-positive cholinergic fibers that extended from the cell body were counted according to Suzuki *et al*. (2004) [52]. We also quantified cholinergic fiber density using the following method: images of AChE-positive cholinergic fibers were captured using the optical conditions described above. First, the background intensity was determined by averaging the values obtained from three non-stained areas. The maximal threshold of fiber intensity was defined as 70% above the background intensity. The total intensity of the AChE-positive fibers was determined in an optical field (500 × 500 pixels/field) and was divided by the number of AchE-positive cholinergic neurons in the same field. Data were collected from four independent fields in a single chamber. All experiments were performed in a blind fashion.

### Quantitative analysis of dendritic spine density

Dendritic spine density was quantified according to previous reports [31,33,56], with a few modifications. Cultured hippocampal neurons were treated with the indicated reagents for 2–3 days and were then fixed using 4% paraformaldehyde/PBS for 20 min. DiI solution (20 mg/ml) was used for labeling dendritic spines. For visualization of F-actin accumulated in the dendritic spines, fixed cells were immunostained with an anti-MAP2 antibody [52], followed by 1 h incubation with TRITC-conjugated phalloidin (1:750 in PBS). We used confocal microscopy (FV-1000, Olympus, Tokyo, Japan) with a 60 × objective lens to capture stacked images of dendritic spines along the z-axis of the imaged cell. The spatial resolution of captured images was 0.1 μm/pixel. The number of spines was quantitatively evaluated at the proximal side of the second dendrite via manual counting using Metamorph software (Universal Imaging, Downingtown, PA). Dendritic spines in slice cultures were labeled by rhodamine-dextran injection (6 mg/ml in a patch pipette) using a single-cell electroporator [57].

### Hippocampal slice cultures

Hippocampal organotypic slice cultures were prepared from B6C3F1 mice (postnatal day 5–7), according to previous reports [58,59], with a few modifications. Briefly, hippocampi were dissected in ice-cold choline-based artificial cerebrospinal fluid (ACSF) (in which NaCl was replaced by Choline-Cl) and were cut transversely into 350 μm-thick slices. The slices were transferred to Millicall CM membrane inserts (Millipore) and were maintained in medium containing 80% modified MEM (described next) with 20% HS. Modified MEM contained 50% MEM, 25% Hanks' Balanced Salt Solution (HBSS), glucose (30.5 mM), CaCl_2 _(1 mM), MgCl_2 _(2 mM), NaHCO_3 _(13 mM), ascorbic acid (0.68 mM), and HEPES (25 mM). The culture medium was half-replaced every 2 days.

### Electrophysiology

The electrophysiological study was carried out as described previously [37,38]. Miniature excitatory synaptic currents (mEPSCs) were recordered in the voltage-clamp mode of the whole-cell patch-clamp technique at room temperature (23–24°C). The recording chamber was continuously perfused at a rate of 0.2 ml/min with an extracellular solution containing 124 mM NaCl, 1.25 mM NaHPO_4_, 5 mM KCl, 33.3 mM glucose, 2 mM CaCl_2_, 1 mM MgCl_2_, 0.1 mM picrotoxin (PTX) (TCI, Tokyo, Japan), 0.5 μM tetorodotoxin (TTX), and 22 mM HEPES, with pH and osmolality adjusted to 7.4 and 310–320 mOsmol with NaOH and sucrose, respectively. The solutions in the patch pipettes contained (in mM) 110 Cs methanesulfonate, 20 CsCl_2_, 20 HEPES, 0.1 spermine, 10 Na-phosphocreatine, 0.6 EGTA-Cs, 0.1 GTP-Na_3_, and 4 ATP-Na_2 _(pH 7.3). All chemicals used in the electrophysiological experiments were obtained from Wako, unless otherwise specified. mEPSCs were analyzed using the MINI ANALYSIS software (Synaptosoft, Decatur, GA) and house-made macro programs.

### Statistical analyses

Typically, significance was determined using the Student's *t*-test after testing for normal distribution. In several multi-bar figures, significance was analyzed by ANOVA followed by a post-hoc comparison. In all graphs, data were presented as means ± standard error of the mean (SEM).

## Methods for additional files

### Fluorescent cleavage assay

Peptides were prepared in an automated peptide synthesizer (PSSM-8, Shimazu, Japan) using Fmoc chemistry. A fluorogenic group (7-methoxycoumarin-4-yl acetyl, MOCAc) and a quenching group (2,4-dinitrophenyl, DNP) were introduced into peptides using (7-methoxy-coumarin-4-yl)-acetic acid (Bachem, Bubendorf, Switzerland) and *N*-alpha-fluorenylmethoxycarbonyl-*N*-epsilon-dinitrophenyl-lysine (Novabiochem, Laufelfingen, Switzerland), respectively [60]. MOCAc- and DNP-conjugated peptides (5 mM) were solved in a buffer containing 0.1% (w/v) Triton X-100, 100 mM HEPES (pH 7.5), and 1 mM CaCl_2_. Furin (10 units/ml; Sigma) was added to the peptide solution and the mixture was incubated at 30°C. The furin digestion was monitored by measuring the fluorescence of the cleaved MOCAc-containing peptide at 393 nm after excitation at 323 nm, using an F-4500 Fluorescence Spectrophotometer (Hitachi, Tokyo, Japan).

### Circular dichroism (CD) measurements

CD measurements were carried out using a spectropolarimeter (model J-820, Jasco, Tokyo, Japan). The spectra were accumulated by performing four scans at a scan rate of 20 nm/min using a time constant of 1.0 sec. CD spectra were recorded at 25°C using a cell with a 1 mm path length. The protein concentration used for all measurements was 21 μM. The concentration of protein samples was estimated using the calculated molar absorption coefficient at 280 nm (ε280 = 32,275 mol^-1^cm^-1^l). The production of proNGF and proNT-3 proteins was carried out as described for CR-proBDNF. Heat-denatured (boiled) CR-proBDNF was prepared by 30 min boiling prior to the application to culture dishes.

## Competing interests

The authors declare that they have no competing interests.

## Authors' contributions

Conceived and designed the experiments: HK KK SS KU GN EZ TH YT AO BL MK. Performed the experiments: HK KK TH SH YT MK. Analyzed the data: HK KK SH TH YT MK. Wrote the paper: HK KK BL MK. All authors read and approved the final manuscript.

## Supplementary Material

Additional file 1 **DNA constructs and proBDNF-specific antibodies were generated for characterization of R125M, R127, and R125/R127 substitutions**. (A) DNA constructs used in the present study (Figs. 1 and 2). The DNA constructs encoded wild-type, single-, and double-mutant BDNF proteins (RR, M, L, ML). The gray, pink, and blue regions indicate the signal peptide, the prodomain, and the mature domain, respectively. The amino acid substitutions caused by the SNPs are depicted by white symbols. (B) Specificity of the proBDNF antibodies. Mouse, rabbit, and chicken antibodies specifically recognized *E. coli*-derived CR-proBDNF (R125M/R127L-BDNF) on Western blot (Figs. 1 and 3), but not proNGF or proNT-3. Ten ng of each recombinant protein was loaded for silver staining and immunoblotting. These antibodies did not recognize matBDNF, matNGF, or matNT-3 (data not shown). Click here for file

Additional file 2 ***E. coli*-derived CR-proBDNF acquires its proper secondary structure and is not able to elicit apoptosis of CGNs cultured in HK medium**. (A) circular dichroism (CD) spectroscopy of refolded *E. coli*-derived CR-proBDNF. The CR-proBDNF (proBDNF_ML_) protein expressed in *E. coli *(Fig. 3A) formed inclusion bodies; therefore, we used urea denaturation for purification followed by refolding of the protein (Methods). The refolded CR-proBDNF exhibited a secondary structure, as assessed using CD spectroscopy. A wavelength scan was acquired from 255–205 nm, before (dashed line) and after (solid line) the refolding reaction. The CD spectrum demonstrates that the resulting proBDNF had a secondary structure with a peak at 218 nm. (B-C) CR-proBDNF is not able to activate TrkB receptor but involves p75^NTR ^for the cell death. After 4 days of culture in HK medium, rat (B) and mouse CGNs (C) were treated with the indicated drug in LK medium. (B) Failure to activate TrkB by CR-proBDNF. A time-course study demonstrated that matBDNF, but not CR-proBDNF, activated TrkB (upper panel). Western blots were performed with anti-TrkB (clone 47, BD Biosciences) and anti-phospho-TrkB (Y490, Cell Signaling) antibodies. (C) The proapoptotic effect of CR-proBDNF is dependent on p75^NTR^. CGNs derived from p75NTR^-/- ^mice were processed for DAPI staining as described in Fig. 3B. The Student's t-test was used and compared to Mock. **P < 0.01. n = 4 independent culture dishes. Results were replicated in at least three independent experiments. Please note that CR-proBDNF did not significantly induce apoptosis in CGNs derived from p75NTR^-/- ^mice. (D) Apoptotic effect of recombinant CR-proBDNF derived from Baculovirus and *E. Coli*. CGNs were cultured, treated with CR-proBDNF, and apoptosis was measured using the LDH-cytotoxic assay, as described in Methods. Heat-denatured CR-proBDNF was used as a control. In multi-bar graphs, ANOVA was followed by post-hoc analysis. **P < 0.01, n = 3 independent coverslips. (E) Absence of a significant effect of CR-proBDNF on the viability of CGNs cultured in HK medium. After 4 days of culture in HK medium, CGNs were treated with *E. coli*-derived CR-proBDNF or matBDNF in HK medium (but not LK medium). Cell viability was assessed using the DAPI staining method 48 h later. n = 3 independent coverslips. Click here for file

Additional file 3 **Two-day treatment with proBDNFML does not affect the number of MAP2-positive neurons**. As described in Fig. 6A, the hippocampal cultures (1 × 10^5 ^cells/cm^2^) were maintained for 3–4 weeks and treated with 100 ng/ml *E. coli*-derived CR-proBDNF or 100 ng/ml matBDNF for two days. Cells were then fixed and stained using an antibody against MAP2 [52]. The number of MAP2-positive neurons was counted as described. (A) Representative images of MAP2-positive neurons. Scale bar, 10 μm. (B) Quantitative analysis of the number of MAP2-positive cells. n = 4 independent culture dishes. Two-day treatment with matBDNF or CR-proBDNF did not appear to affect the overall morphology of mature hippocampal neurons. Click here for file

Additional file 4 **M, L, and ML variants are resistant to furin, revealed by an in vitro protease digestion assay**. Ten-amino-acid polypeptides that contained the cleavage site of proBDNF with the indicated amino acid residues were conjugated with MOCAc and DNP at the N- and C-termini, respectively [60]: the latter quenches the fluorescence in the absence of peptide cleavage. The peptides were incubated with the widely distributed prohormone convertase, furin (10 units/ml) [2]. Cleavage of wild-type BDNF (BDNF_RR_) peptide by furin would separate the fluorogenic group from the quencher and would thus generate a measurable fluorescent signal. A time-course study revealed a gradual increase in fluorescence after the incubation of the BDNFRR peptide with furin [2], which reached a maximum at 4 h (black circles). In contrast, R125M-BDNF (BDNF_M_), R127L-BDNF (BDNF_L_), and R125M/R127L-BDNF (BDNF_ML_) peptides did not elicit a significant increase in fluorescence (red, blue, and purple circles, respectively). Mass spectrometric analysis confirmed that the BDNF_RR _peptide was precisely cleaved at the processing site (data not shown). Click here for file
