# Supplementary figures and images for: Multiple functions of precursor BDNF to CNS neurons: negative regulation of neurite growth, spine formation and cell survival
Source: Mol Brain. 2009 Aug 13;2:27. doi: 10.1186/1756-6606-2-27 (PMC2743674; doi:10.1186/1756-6606-2-27)

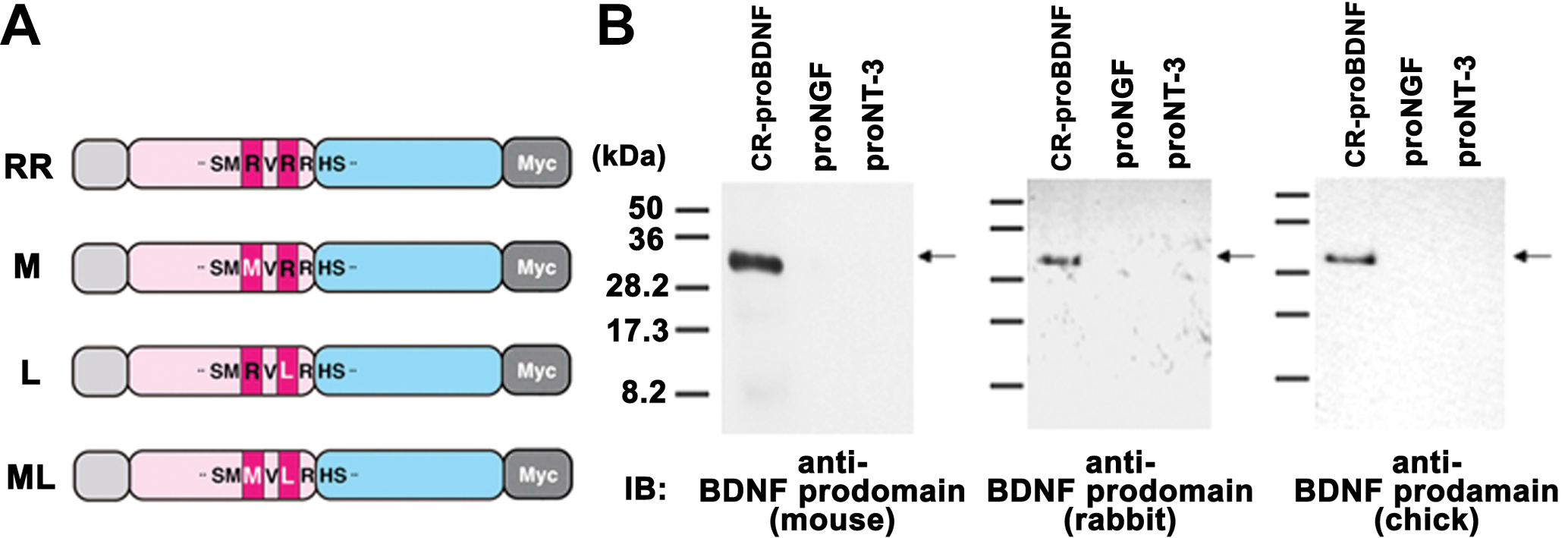

Supplement: Additional file 1 — DNA constructs and proBDNF-specific antibodies were generated for characterization of R125M, R127, and R125/R127 substitutions. (A) DNA constructs used in the present study (Figs. 1 and 2). The DNA constructs encoded wild-type, single-, and double-mutant BDNF proteins (RR, M, L, ML). The gray, pink, and blue regions indicate the signal peptide, the prodomain, and the mature domain, respectively. The amino acid substitutions caused by the SNPs are depicted by white symbols. (B) Specificity of the proBDNF antibodies. Mouse, rabbit, and chicken antibodies specifically recognized E. coli-derived CR-proBDNF (R125M/R127L-BDNF) on Western blot (Figs. 1 and 3), but not proNGF or proNT-3. Ten ng of each recombinant protein was loaded for silver staining and immunoblotting. These antibodies did not recognize matBDNF, matNGF, or matNT-3 (data not shown). [file 1756-6606-2-27-S1.tiff]

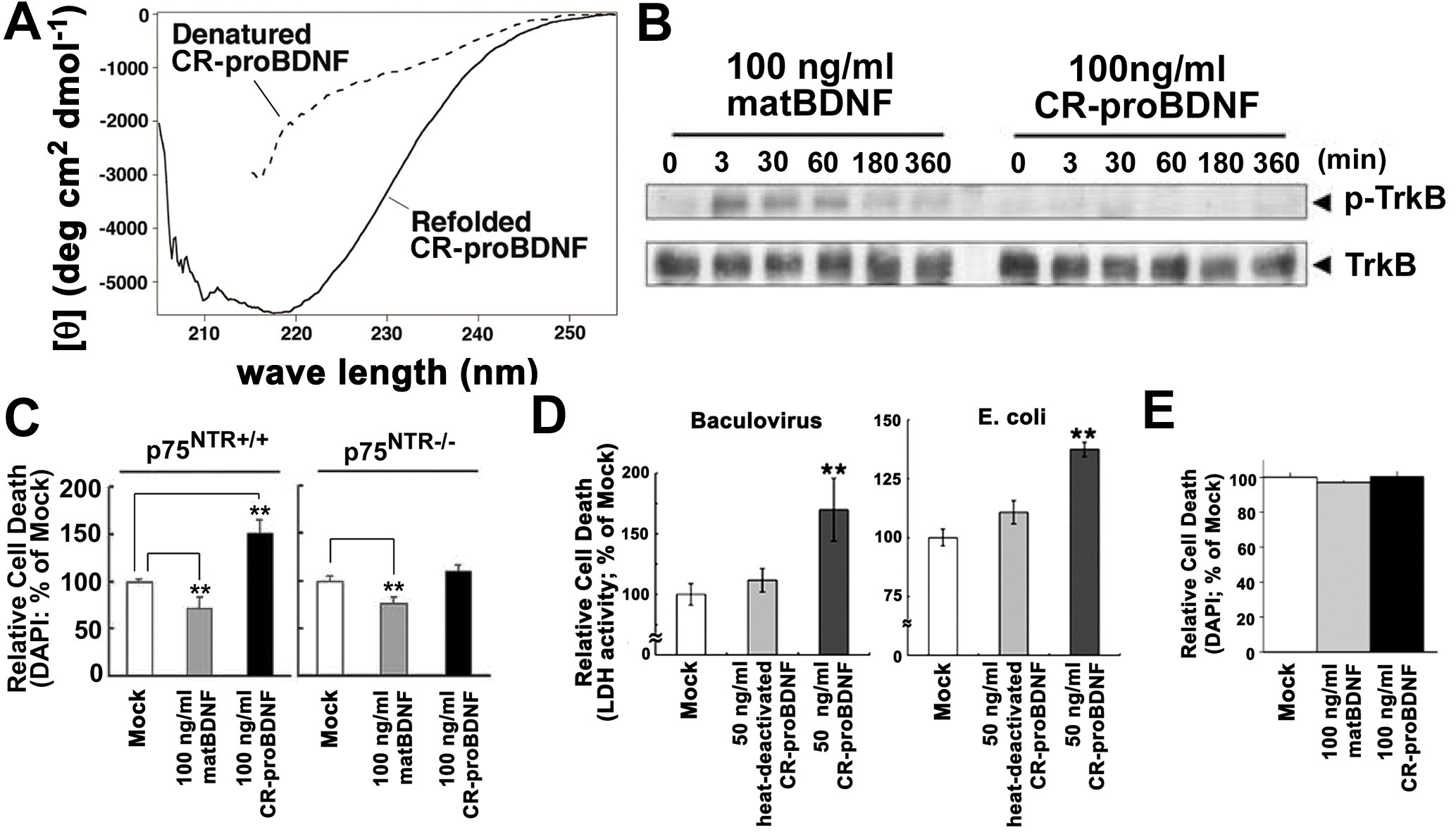

Supplement: Additional file 2 — E. coli-derived CR-proBDNF acquires its proper secondary structure and is not able to elicit apoptosis of CGNs cultured in HK medium. (A) circular dichroism (CD) spectroscopy of refolded E. coli-derived CR-proBDNF. The CR-proBDNF (proBDNFML) protein expressed in E. coli (Fig. 3A) formed inclusion bodies; therefore, we used urea denaturation for purification followed by refolding of the protein (Methods). The refolded CR-proBDNF exhibited a secondary structure, as assessed using CD spectroscopy. A wavelength scan was acquired from 255–205 nm, before (dashed line) and after (solid line) the refolding reaction. The CD spectrum demonstrates that the resulting proBDNF had a secondary structure with a peak at 218 nm. (B-C) CR-proBDNF is not able to activate TrkB receptor but involves p75NTR for the cell death. After 4 days of culture in HK medium, rat (B) and mouse CGNs (C) were treated with the indicated drug in LK medium. (B) Failure to activate TrkB by CR-proBDNF. A time-course study demonstrated that matBDNF, but not CR-proBDNF, activated TrkB (upper panel). Western blots were performed with anti-TrkB (clone 47, BD Biosciences) and anti-phospho-TrkB (Y490, Cell Signaling) antibodies. (C) The proapoptotic effect of CR-proBDNF is dependent on p75NTR. CGNs derived from p75NTR-/- mice were processed for DAPI staining as described in Fig. 3B. The Student's t-test was used and compared to Mock. **P < 0.01. n = 4 independent culture dishes. Results were replicated in at least three independent experiments. Please note that CR-proBDNF did not significantly induce apoptosis in CGNs derived from p75NTR-/- mice. (D) Apoptotic effect of recombinant CR-proBDNF derived from Baculovirus and E. Coli. CGNs were cultured, treated with CR-proBDNF, and apoptosis was measured using the LDH-cytotoxic assay, as described in Methods. Heat-denatured CR-proBDNF was used as a control. In multi-bar graphs, ANOVA was followed by post-hoc analysis. **P < 0.01, n = 3 independent coverslips. (E) Abs [file 1756-6606-2-27-S2.tiff]

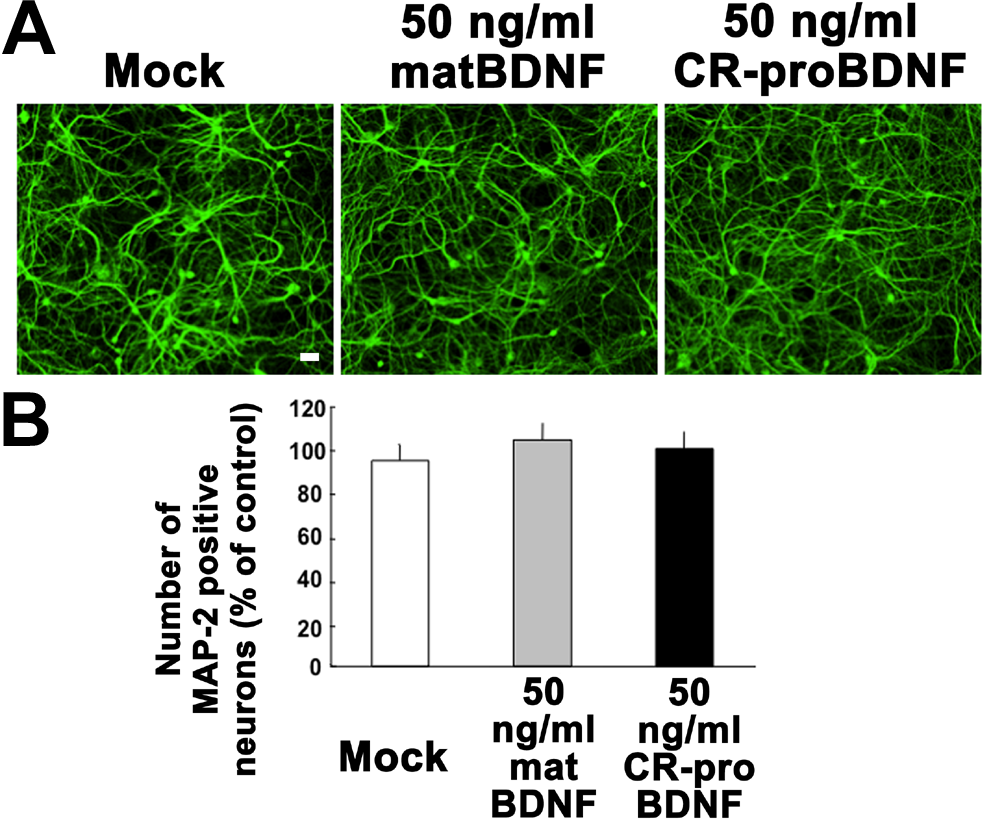

Supplement: Additional file 3 — Two-day treatment with proBDNFML does not affect the number of MAP2-positive neurons. As described in Fig. 6A, the hippocampal cultures (1 × 10^5^ cells/cm2) were maintained for 3–4 weeks and treated with 100 ng/ml E. coli-derived CR-proBDNF or 100 ng/ml matBDNF for two days. Cells were then fixed and stained using an antibody against MAP2 [52]. The number of MAP2-positive neurons was counted as described. (A) Representative images of MAP2-positive neurons. Scale bar, 10 μm. (B) Quantitative analysis of the number of MAP2-positive cells. n = 4 independent culture dishes. Two-day treatment with matBDNF or CR-proBDNF did not appear to affect the overall morphology of mature hippocampal neurons. [file 1756-6606-2-27-S3.tiff]

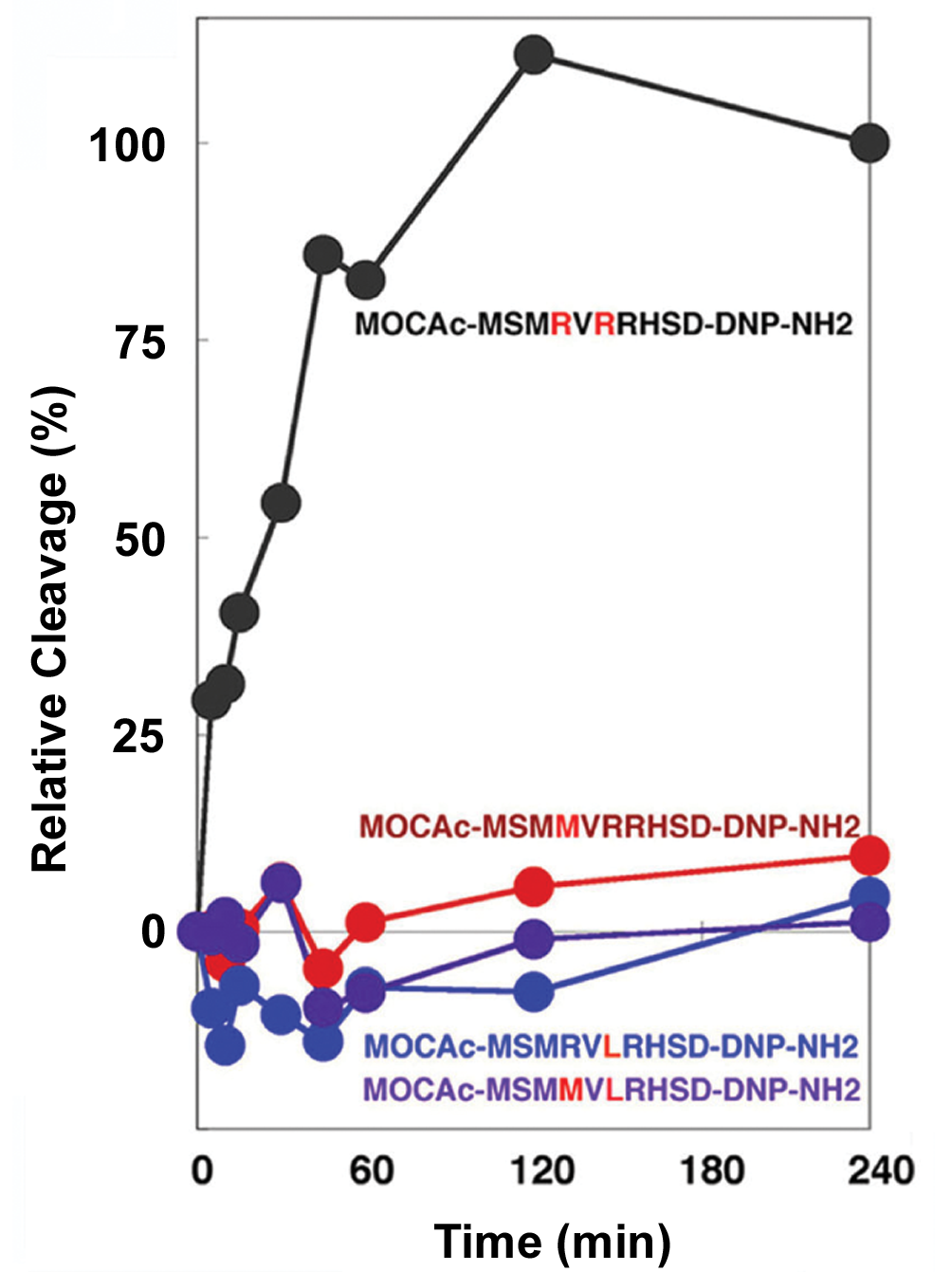

Supplement: Additional file 4 — M, L, and ML variants are resistant to furin, revealed by an in vitro protease digestion assay. Ten-amino-acid polypeptides that contained the cleavage site of proBDNF with the indicated amino acid residues were conjugated with MOCAc and DNP at the N- and C-termini, respectively [60]: the latter quenches the fluorescence in the absence of peptide cleavage. The peptides were incubated with the widely distributed prohormone convertase, furin (10 units/ml) [2]. Cleavage of wild-type BDNF (BDNFRR) peptide by furin would separate the fluorogenic group from the quencher and would thus generate a measurable fluorescent signal. A time-course study revealed a gradual increase in fluorescence after the incubation of the BDNFRR peptide with furin [2], which reached a maximum at 4 h (black circles). In contrast, R125M-BDNF (BDNFM), R127L-BDNF (BDNFL), and R125M/R127L-BDNF (BDNFML) peptides did not elicit a significant increase in fluorescence (red, blue, and purple circles, respectively). Mass spectrometric analysis confirmed that the BDNFRR peptide was precisely cleaved at the processing site (data not shown). [file 1756-6606-2-27-S4.tiff]
